# 1-{2-Benz­yloxy-2-[4-(morpholin-4-yl)phen­yl]eth­yl}-1*H*-benzimidazole

**DOI:** 10.1107/S1600536812051306

**Published:** 2012-12-22

**Authors:** Özden Özel Güven, Seval Çapanlar, Philip D. F. Adler, Simon J. Coles, Tuncer Hökelek

**Affiliations:** a Department of Chemistry, Bülent Ecevit University, 67100 Zonguldak, Turkey; b Department of Chemistry, Southampton University, SO17 1BJ Southampton, England; c Department of Physics, Hacettepe University, 06800 Beytepe, Ankara, Turkey

## Abstract

In the title compound, C_26_H_27_N_3_O_2_, the morpholine ring adopts a chair conformation. The benzene and phenyl rings are inclined to the benzimidazole mean plane by 7.28 (6) and 61.45 (4)°, respectively. In the crystal, pairs of weak C—H⋯O hydrogen bonds link the mol­ecules into inversion dimers. These dimers are further connected *via* weak C—H⋯N hydrogen bonds. A weak C—H⋯π inter­action is also observed.

## Related literature

For general background to the biological activity of benzimidazole derivatives, see: Özel Güven *et al.* (2007*a* ▶,*b* ▶). For related structures, see: Caira *et al.* (2004 ▶); Freer *et al.* (1986 ▶); Özel Güven *et al.* (2008*a* ▶,*b* ▶,*c* ▶); Peeters *et al.* (1979*a* ▶,*b* ▶, 1996 ▶). For hydrogen-bonding motifs, see: Bernstein *et al.* (1995 ▶). For ring puckering parameters, see: Cremer & Pople (1975 ▶).
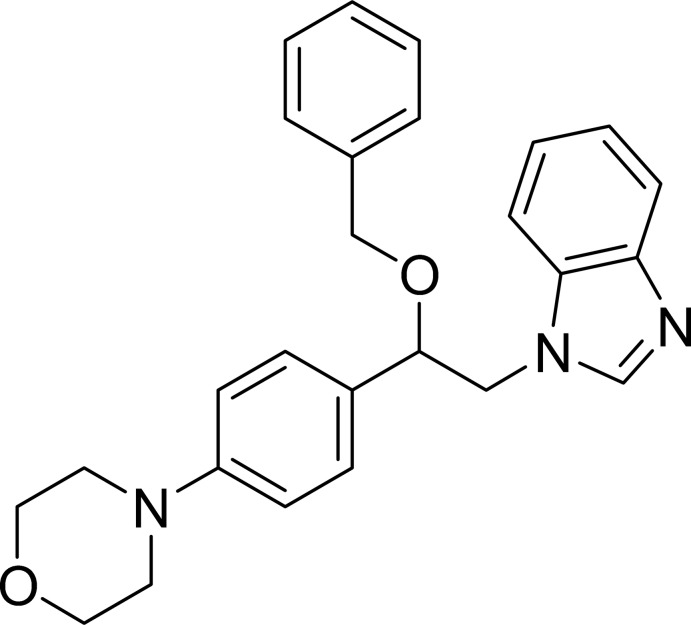


## Experimental

### 

#### Crystal data

C_26_H_27_N_3_O_2_
*M* *_r_* = 413.51
Triclinic, 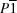

*a* = 9.4719 (3) Å
*b* = 10.5057 (3) Å
*c* = 11.8110 (4) Å
α = 96.824 (3)°
β = 108.953 (4)°
γ = 98.312 (3)°
*V* = 1082.51 (7) Å^3^
*Z* = 2
Mo *K*α radiation
μ = 0.08 mm^−1^
*T* = 100 K
0.32 × 0.25 × 0.22 mm

#### Data collection

Rigaku Saturn724+ diffractometer
13756 measured reflections
4972 independent reflections
3736 reflections with *I* > 2σ(*I*)
*R*_int_ = 0.043
3 standard reflections every 2 min intensity decay: 1%

#### Refinement

*R*[*F* ^2^ > 2σ(*F* ^2^)] = 0.051
*wR*(*F* ^2^) = 0.135
*S* = 1.06
4972 reflections
280 parameters
H-atom parameters constrained
Δρ_max_ = 0.63 e Å^−3^
Δρ_min_ = −0.24 e Å^−3^

### 

Data collection: *CrystalClear-SM Expert* (Rigaku, 2011 ▶); cell refinement: *CrystalClear-SM Expert*; data reduction: *CrystalClear-SM Expert*; program(s) used to solve structure: *SHELXS97* (Sheldrick, 2008 ▶); program(s) used to refine structure: *SHELXL97* (Sheldrick, 2008 ▶); molecular graphics: *ORTEP-3 for Windows* (Farrugia, 2012 ▶); software used to prepare material for publication: *WinGX* (Farrugia, 2012 ▶) and *PLATON* (Spek, 2009 ▶).

## Supplementary Material

Crystal structure: contains datablock(s) I, global. DOI: 10.1107/S1600536812051306/cv5371sup1.cif

Structure factors: contains datablock(s) I. DOI: 10.1107/S1600536812051306/cv5371Isup2.hkl

Supplementary material file. DOI: 10.1107/S1600536812051306/cv5371Isup3.cml

Additional supplementary materials:  crystallographic information; 3D view; checkCIF report

## Figures and Tables

**Table 1:** Hydrogen-bond geometry (Å, °) *Cg* is the centroid of the C4–C9 benzene ring.

*D*—H⋯*A*	*D*—H	H⋯*A*	*D*⋯*A*	*D*—H⋯*A*
C5—H5⋯O2^i^	0.93	2.58	3.478 (2)	163
C6—H6⋯N3^ii^	0.93	2.56	3.439 (2)	159
C2—H2*A*⋯*Cg* ^iii^	0.97	2.66	3.451 (2)	139

Symmetry codes: (i) 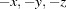; (ii) 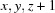; (iii) 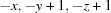.

**Fig. 1.**
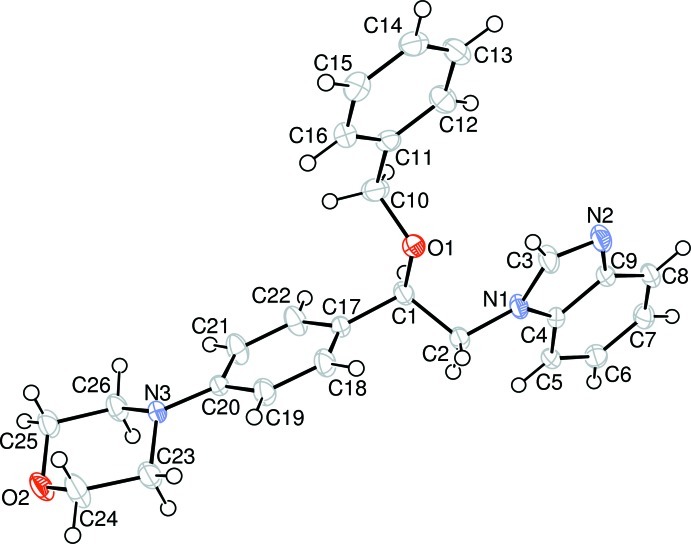
The molecular structure of the title compound with the atom-numbering scheme. Displacement ellipsoids are drawn at the 50% probability level.

**Fig. 2.**
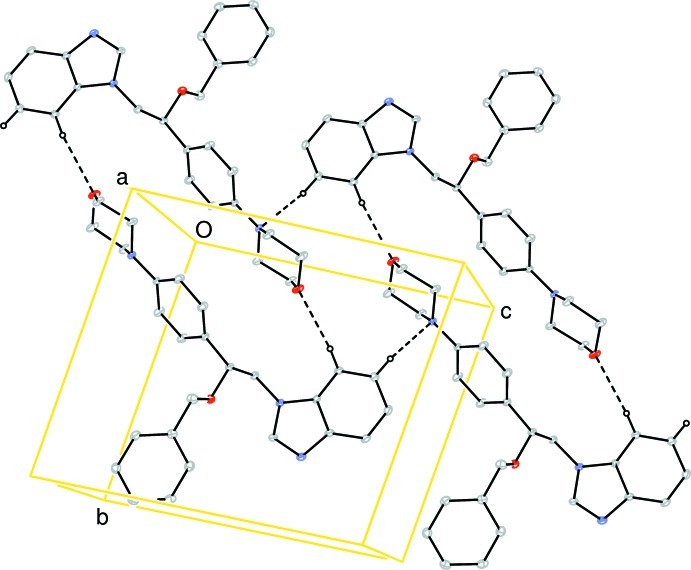
A partial packing diagram. C–H···O and C–H···N hydrogen bonds are shown as dashed lines. Hydrogen atoms not involved in hydrogen bonding have been omitted for clarity.

## supplementary crystallographic information

### Comment

The azole compounds possessing an imidazole or triazole ring (such as econazole, miconazole, ketoconazole, fluconazole and itraconazole) have been known as antifungal agents and used in clinics. Similar structures possessing benzimidazole ring in place of imidazole ring of miconazole and econazole have been reported to show antibacterial activity higher than antifungal activity (Özel Güven *et al.*, 2007*a*,*b*). The crystal structures of econazole (Freer *et al.*, 1986), miconazole (Peeters *et al.*, 1979*a*), ketoconazole (Peeters *et al.*, 1979*b*), fluconazole (Caira *et al.*, 2004) and itraconazole (Peeters *et al.*, 1996) have been reported, previously. Crystal structures of similar ether compounds having benzimidazole ring have been reported (Özel Güven *et al.*, 2008*a*,*b*,*c*). Herewith we report the crystal structure of the title compound (I), which is a new benzimidazole derivative.

In (I) (Fig. 1), the bond lengths and angles are within normal ranges. The benzimidazole [A (N1/N2/C3–C9)] ring system is approximately planar with a maximum deviation of -0.010 (2) Å for atom C2 and its mean plane is oriented with respect to the benzene [B (C11–C16)] and phenyl [C (C17–C22)] rings at dihedral angles of A/B = 7.28 (6) and A/C = 61.45 (4) °. The dihedral angle between benzene and phenyl rings is B/C = 54.96 (5)°. Atom C10 is -0.008 (2) Å away from the plane of the benzene ring and atoms C1 and N3 are 0.056 (2) and 0.076 (2) Å away from the plane of the phenyl ring. The morpholine ring D (C23–C26/O2/N3) is not planar, but adopting a chair conformation with puckering parameters (Cremer & Pople, 1975) Q_T_ = 1.060 (5)Å, φ = 34.3 (2)° and θ = 57.3 (1)°.

In the crystal structure, weak intermolecular C—H···O hydrogen bonds (Table 1) link the molecules into centrosymmetric R_2_^2^(28) dimers (Bernstein *et al.*, 1995). These dimers are further connected *via* weak intermolecular C—H···N hydrogen bonds (Table 1), linking the molecules into centrosymmetric R_4_^4^(16) dimers, to form a 2-D network (Fig. 2). A weak C—H···π interaction (Table 1) is also observed.

### Experimental

The title compound, (I), was synthesized by the reaction of 2-(1*H*-benzimidazol-1-yl)-1-(4-morpholinophenyl)ethanol with aryl halide using sodium hydrate. NaH (0.025 g, 0.618 mmol) was added to a solution of alcohol (0.2 g, 0.618 mmol) in DMF (4 ml) in small fractions. After stirring the mixture a few minutes, benzyl bromide (0.073 ml, 0.618 mmol) was added dropwise. Then, the reaction mixture was stirred additional 3 h at room temperature. The reaction was stopped by adding a small amount of methyl alcohol. After evaporation of the solvent, dichloromethane was added to the reaction mixture and extracted with water. The organic phase was separated and dried with anhydrous magnesium sulfate, then evaporated to dryness. The residue was purified by column chromatography using chloroform and crystallized from DMSO:H_2_O (1:1) to obtain colorless crystals suitable for X-ray analysis (yield; 0.089 g, 35%).

### Refinement

H atoms were positioned geometrically with C—H = 0.98, 0.93 and 0.97 Å for methine, aromatic and methylene H, respectively, and constrained to ride on their parent atoms, with *U*_iso_(H) = 1.2 *U*_eq_(C). The highest residual electron density was found 1.10 Å from H1 and the deepest hole 0.59 Å from O1.

### Crystal data

**Table:**

C_26_H_27_N_3_O_2_	*Z* = 2
*M**_r_* = 413.51	*F*(000) = 440
Triclinic, *P*1	*D*_x_ = 1.269 Mg m^−^^3^
Hall symbol: -P 1	Mo *K*α radiation, λ = 0.71073 Å
*a* = 9.4719 (3) Å	Cell parameters from 11428 reflections
*b* = 10.5057 (3) Å	θ = 3.0–27.6°
*c* = 11.8110 (4) Å	µ = 0.08 mm^−^^1^
α = 96.824 (3)°	*T* = 100 K
β = 108.953 (4)°	Prism, colourless
γ = 98.312 (3)°	0.32 × 0.25 × 0.22 mm
*V* = 1082.51 (7) Å^3^	

### Data collection

**Table:**

Rigaku Saturn724+ diffractometer	*R*_int_ = 0.043
Radiation source: fine-focus sealed tube	θ_max_ = 27.5°, θ_min_ = 3.1°
Graphite monochromator	*h* = −12→12
profile data from ω–scans	*k* = −13→13
13756 measured reflections	*l* = −15→15
4972 independent reflections	3 standard reflections every 2 min
3736 reflections with *I* > 2σ(*I*)	intensity decay: 1%

### Refinement

**Table:**

Refinement on *F*^2^	Primary atom site location: structure-invariant direct methods
Least-squares matrix: full	Secondary atom site location: difference Fourier map
*R*[*F*^2^ > 2σ(*F*^2^)] = 0.051	Hydrogen site location: inferred from neighbouring sites
*wR*(*F*^2^) = 0.135	H-atom parameters constrained
*S* = 1.06	*w* = 1/[σ^2^(*F*_o_^2^) + (0.0577*P*)^2^ + 0.355*P*] where *P* = (*F*_o_^2^ + 2*F*_c_^2^)/3
4972 reflections	(Δ/σ)_max_ < 0.001
280 parameters	Δρ_max_ = 0.63 e Å^−^^3^
0 restraints	Δρ_min_ = −0.24 e Å^−^^3^

### Special details

**Table:**

Geometry. All esds (except the esd in the dihedral angle between two l.s. planes) are estimated using the full covariance matrix. The cell esds are taken into account individually in the estimation of esds in distances, angles and torsion angles; correlations between esds in cell parameters are only used when they are defined by crystal symmetry. An approximate (isotropic) treatment of cell esds is used for estimating esds involving l.s. planes.
Refinement. Refinement of F^2^ against ALL reflections. The weighted R-factor wR and goodness of fit S are based on F^2^, conventional R-factors R are based on F, with F set to zero for negative F^2^. The threshold expression of F^2^ > 2sigma(F^2^) is used only for calculating R-factors(gt) etc. and is not relevant to the choice of reflections for refinement. R-factors based on F^2^ are statistically about twice as large as those based on F, and R- factors based on ALL data will be even larger.

### Fractional atomic coordinates and isotropic or equivalent isotropic displacement parameters (Å^2^)

**Table:**

	*x*	*y*	*z*	*U*_iso_*/*U*_eq_	
O1	0.28084 (13)	0.57424 (10)	0.27381 (10)	0.0245 (3)	
O2	−0.08746 (15)	−0.09871 (11)	−0.38295 (11)	0.0336 (3)	
N1	0.18512 (16)	0.52783 (12)	0.47120 (12)	0.0239 (3)	
N2	0.28757 (19)	0.70971 (13)	0.61301 (13)	0.0312 (3)	
N3	0.03315 (15)	0.10262 (12)	−0.17348 (11)	0.0203 (3)	
C1	0.24066 (19)	0.44368 (15)	0.28999 (14)	0.0240 (3)	
H1	0.3308	0.4185	0.3444	0.029*	
C2	0.12280 (19)	0.44506 (15)	0.35127 (14)	0.0243 (3)	
H2A	0.0368	0.4768	0.3008	0.029*	
H2B	0.0864	0.3566	0.3593	0.029*	
C3	0.2357 (2)	0.65983 (15)	0.49714 (15)	0.0296 (4)	
H3	0.2334	0.7103	0.4372	0.036*	
C4	0.20304 (18)	0.48813 (15)	0.58148 (13)	0.0211 (3)	
C5	0.16909 (19)	0.36691 (15)	0.61283 (14)	0.0240 (3)	
H5	0.1268	0.2914	0.5544	0.029*	
C6	0.2014 (2)	0.36446 (16)	0.73509 (15)	0.0288 (4)	
H6	0.1793	0.2854	0.7596	0.035*	
C7	0.2668 (2)	0.47816 (17)	0.82337 (15)	0.0303 (4)	
H7	0.2874	0.4726	0.9050	0.036*	
C8	0.3011 (2)	0.59754 (16)	0.79188 (15)	0.0298 (4)	
H8	0.3452	0.6724	0.8509	0.036*	
C9	0.26797 (19)	0.60318 (15)	0.66909 (15)	0.0244 (3)	
C10	0.4207 (2)	0.59640 (17)	0.25038 (18)	0.0319 (4)	
H10A	0.4128	0.5376	0.1778	0.038*	
H10B	0.5034	0.5810	0.3182	0.038*	
C11	0.45016 (18)	0.73490 (16)	0.23337 (15)	0.0253 (3)	
C12	0.5176 (2)	0.83670 (17)	0.33221 (16)	0.0313 (4)	
H12	0.5461	0.8184	0.4108	0.038*	
C13	0.5426 (2)	0.96468 (17)	0.31489 (16)	0.0317 (4)	
H13	0.5868	1.0319	0.3817	0.038*	
C14	0.5024 (2)	0.99292 (17)	0.19901 (16)	0.0293 (4)	
H14	0.5206	1.0789	0.1874	0.035*	
C15	0.4350 (2)	0.89280 (18)	0.10032 (16)	0.0320 (4)	
H15	0.4065	0.9114	0.0219	0.038*	
C16	0.4096 (2)	0.76504 (17)	0.11782 (16)	0.0303 (4)	
H16	0.3645	0.6982	0.0507	0.036*	
C17	0.18236 (19)	0.34981 (15)	0.17011 (14)	0.0224 (3)	
C18	0.0535 (2)	0.36116 (17)	0.07608 (16)	0.0307 (4)	
H18	−0.0005	0.4254	0.0888	0.037*	
C19	0.0033 (2)	0.27977 (17)	−0.03558 (15)	0.0296 (4)	
H19	−0.0826	0.2910	−0.0968	0.036*	
C20	0.07978 (18)	0.18079 (14)	−0.05807 (13)	0.0199 (3)	
C21	0.2087 (2)	0.16989 (17)	0.03657 (16)	0.0326 (4)	
H21	0.2625	0.1051	0.0250	0.039*	
C22	0.2586 (2)	0.25367 (17)	0.14774 (15)	0.0320 (4)	
H22	0.3459	0.2444	0.2087	0.038*	
C23	−0.1301 (2)	0.06684 (18)	−0.24194 (16)	0.0332 (4)	
H23A	−0.1756	0.1436	−0.2386	0.040*	
H23B	−0.1772	0.0031	−0.2055	0.040*	
C24	−0.1588 (2)	0.0102 (2)	−0.37283 (17)	0.0406 (5)	
H24A	−0.2675	−0.0169	−0.4157	0.049*	
H24B	−0.1206	0.0772	−0.4112	0.049*	
C25	0.0716 (2)	−0.05690 (18)	−0.32175 (16)	0.0332 (4)	
H25A	0.1098	0.0112	−0.3590	0.040*	
H25B	0.1220	−0.1297	−0.3305	0.040*	
C26	0.1096 (2)	−0.00560 (17)	−0.18839 (15)	0.0296 (4)	
H26A	0.0774	−0.0750	−0.1495	0.036*	
H26B	0.2187	0.0238	−0.1497	0.036*	

### Atomic displacement parameters (Å^2^)

**Table:**

	*U*^11^	*U*^22^	*U*^33^	*U*^12^	*U*^13^	*U*^23^
O1	0.0256 (6)	0.0211 (6)	0.0273 (6)	0.0030 (5)	0.0113 (5)	0.0010 (5)
O2	0.0349 (7)	0.0277 (6)	0.0291 (7)	0.0044 (5)	0.0054 (5)	−0.0115 (5)
N1	0.0350 (8)	0.0173 (6)	0.0204 (7)	0.0025 (5)	0.0133 (6)	−0.0004 (5)
N2	0.0486 (10)	0.0190 (7)	0.0279 (8)	0.0018 (6)	0.0198 (7)	−0.0012 (6)
N3	0.0219 (7)	0.0198 (6)	0.0177 (6)	0.0028 (5)	0.0071 (5)	−0.0013 (5)
C1	0.0287 (9)	0.0198 (8)	0.0213 (8)	0.0024 (6)	0.0085 (6)	−0.0009 (6)
C2	0.0298 (9)	0.0210 (8)	0.0210 (8)	0.0004 (6)	0.0110 (7)	−0.0011 (6)
C3	0.0475 (11)	0.0171 (8)	0.0273 (9)	0.0044 (7)	0.0188 (8)	0.0010 (6)
C4	0.0234 (8)	0.0207 (7)	0.0199 (7)	0.0027 (6)	0.0108 (6)	−0.0006 (6)
C5	0.0273 (9)	0.0186 (7)	0.0249 (8)	−0.0005 (6)	0.0116 (7)	−0.0013 (6)
C6	0.0368 (10)	0.0241 (8)	0.0286 (9)	0.0022 (7)	0.0164 (7)	0.0058 (7)
C7	0.0373 (10)	0.0336 (9)	0.0202 (8)	0.0032 (8)	0.0127 (7)	0.0023 (7)
C8	0.0371 (10)	0.0252 (8)	0.0238 (8)	−0.0011 (7)	0.0129 (7)	−0.0056 (7)
C9	0.0285 (9)	0.0186 (8)	0.0263 (8)	0.0003 (6)	0.0136 (7)	−0.0019 (6)
C10	0.0268 (9)	0.0280 (9)	0.0419 (10)	0.0021 (7)	0.0153 (8)	0.0044 (7)
C11	0.0181 (8)	0.0262 (8)	0.0312 (9)	0.0023 (6)	0.0099 (6)	0.0019 (7)
C12	0.0299 (9)	0.0330 (9)	0.0247 (9)	0.0044 (7)	0.0030 (7)	0.0025 (7)
C13	0.0273 (9)	0.0262 (9)	0.0334 (9)	0.0015 (7)	0.0044 (7)	−0.0040 (7)
C14	0.0246 (9)	0.0249 (8)	0.0403 (10)	0.0046 (7)	0.0134 (7)	0.0067 (7)
C15	0.0344 (10)	0.0351 (10)	0.0295 (9)	0.0070 (8)	0.0145 (8)	0.0070 (7)
C16	0.0308 (10)	0.0303 (9)	0.0277 (9)	−0.0011 (7)	0.0135 (7)	−0.0041 (7)
C17	0.0260 (8)	0.0205 (7)	0.0200 (8)	−0.0010 (6)	0.0109 (6)	−0.0007 (6)
C18	0.0289 (9)	0.0310 (9)	0.0301 (9)	0.0102 (7)	0.0097 (7)	−0.0063 (7)
C19	0.0247 (9)	0.0345 (9)	0.0258 (9)	0.0096 (7)	0.0054 (7)	−0.0044 (7)
C20	0.0229 (8)	0.0178 (7)	0.0186 (7)	0.0002 (6)	0.0093 (6)	0.0004 (6)
C21	0.0371 (10)	0.0304 (9)	0.0267 (9)	0.0168 (8)	0.0050 (7)	−0.0036 (7)
C22	0.0345 (10)	0.0326 (9)	0.0221 (8)	0.0122 (8)	0.0010 (7)	−0.0023 (7)
C23	0.0263 (9)	0.0376 (10)	0.0288 (9)	0.0067 (8)	0.0055 (7)	−0.0090 (7)
C24	0.0395 (11)	0.0433 (11)	0.0270 (9)	0.0153 (9)	−0.0011 (8)	−0.0123 (8)
C25	0.0326 (10)	0.0333 (9)	0.0296 (9)	0.0076 (8)	0.0102 (7)	−0.0097 (7)
C26	0.0302 (9)	0.0279 (9)	0.0265 (9)	0.0099 (7)	0.0061 (7)	−0.0057 (7)

### Geometric parameters (Å, º)

**Table:**

O1—C1	1.4218 (19)	C11—C12	1.391 (2)
O1—C10	1.432 (2)	C12—H12	0.9300
O2—C24	1.423 (2)	C13—C12	1.382 (2)
O2—C25	1.418 (2)	C13—H13	0.9300
N1—C2	1.4584 (19)	C14—C13	1.377 (3)
N1—C3	1.363 (2)	C14—C15	1.380 (2)
N1—C4	1.380 (2)	C14—H14	0.9300
N2—C3	1.307 (2)	C15—H15	0.9300
N2—C9	1.386 (2)	C16—C11	1.381 (2)
N3—C20	1.4020 (18)	C16—C15	1.381 (2)
N3—C23	1.461 (2)	C16—H16	0.9300
N3—C26	1.4562 (19)	C17—C18	1.389 (2)
C1—C2	1.516 (2)	C17—C22	1.372 (2)
C1—C17	1.512 (2)	C18—H18	0.9300
C1—H1	0.9800	C19—C18	1.380 (2)
C2—H2A	0.9700	C19—H19	0.9300
C2—H2B	0.9700	C20—C19	1.398 (2)
C3—H3	0.9300	C20—C21	1.392 (2)
C5—C4	1.390 (2)	C21—H21	0.9300
C5—C6	1.380 (2)	C22—C21	1.387 (2)
C5—H5	0.9300	C22—H22	0.9300
C6—H6	0.9300	C23—C24	1.510 (2)
C7—C6	1.403 (2)	C23—H23A	0.9700
C7—H7	0.9300	C23—H23B	0.9700
C8—C7	1.374 (2)	C24—H24A	0.9700
C8—C9	1.391 (2)	C24—H24B	0.9700
C8—H8	0.9300	C25—H25A	0.9700
C9—C4	1.409 (2)	C25—H25B	0.9700
C10—H10A	0.9700	C26—C25	1.508 (2)
C10—H10B	0.9700	C26—H26A	0.9700
C11—C10	1.491 (2)	C26—H26B	0.9700
			
C1—O1—C10	112.15 (12)	C12—C13—H13	119.9
C25—O2—C24	108.49 (13)	C14—C13—C12	120.23 (16)
C3—N1—C2	127.13 (14)	C14—C13—H13	119.9
C3—N1—C4	106.11 (13)	C13—C14—C15	119.58 (16)
C4—N1—C2	126.76 (13)	C13—C14—H14	120.2
C3—N2—C9	104.06 (13)	C15—C14—H14	120.2
C20—N3—C23	117.52 (12)	C14—C15—C16	120.10 (16)
C20—N3—C26	118.08 (13)	C14—C15—H15	119.9
C26—N3—C23	111.50 (13)	C16—C15—H15	119.9
O1—C1—C2	106.14 (12)	C11—C16—H16	119.5
O1—C1—C17	111.26 (12)	C15—C16—C11	121.07 (16)
O1—C1—H1	108.9	C15—C16—H16	119.5
C2—C1—H1	108.9	C18—C17—C1	121.39 (14)
C17—C1—C2	112.51 (13)	C22—C17—C1	121.31 (15)
C17—C1—H1	108.9	C22—C17—C18	117.24 (14)
N1—C2—C1	111.66 (13)	C17—C18—H18	119.1
N1—C2—H2A	109.3	C19—C18—C17	121.85 (15)
N1—C2—H2B	109.3	C19—C18—H18	119.1
C1—C2—H2A	109.3	C18—C19—C20	120.98 (16)
C1—C2—H2B	109.3	C18—C19—H19	119.5
H2A—C2—H2B	107.9	C20—C19—H19	119.5
N1—C3—H3	122.7	C19—C20—N3	120.82 (14)
N2—C3—N1	114.56 (15)	C21—C20—N3	122.19 (14)
N2—C3—H3	122.7	C21—C20—C19	116.86 (14)
N1—C4—C5	132.71 (14)	C20—C21—H21	119.3
N1—C4—C9	104.98 (13)	C22—C21—C20	121.35 (15)
C5—C4—C9	122.31 (14)	C22—C21—H21	119.3
C4—C5—H5	121.7	C17—C22—C21	121.70 (16)
C6—C5—C4	116.56 (14)	C17—C22—H22	119.1
C6—C5—H5	121.7	C21—C22—H22	119.1
C5—C6—C7	121.73 (16)	N3—C23—C24	110.50 (14)
C5—C6—H6	119.1	N3—C23—H23A	109.5
C7—C6—H6	119.1	N3—C23—H23B	109.5
C6—C7—H7	119.3	C24—C23—H23A	109.5
C8—C7—C6	121.44 (15)	C24—C23—H23B	109.5
C8—C7—H7	119.3	H23A—C23—H23B	108.1
C7—C8—C9	118.07 (15)	O2—C24—C23	112.00 (15)
C7—C8—H8	121.0	O2—C24—H24A	109.2
C9—C8—H8	121.0	O2—C24—H24B	109.2
N2—C9—C4	110.28 (14)	C23—C24—H24A	109.2
N2—C9—C8	129.83 (15)	C23—C24—H24B	109.2
C8—C9—C4	119.89 (15)	H24A—C24—H24B	107.9
O1—C10—C11	107.45 (13)	O2—C25—C26	111.75 (14)
O1—C10—H10A	110.2	O2—C25—H25A	109.3
O1—C10—H10B	110.2	O2—C25—H25B	109.3
C11—C10—H10A	110.2	C26—C25—H25A	109.3
C11—C10—H10B	110.2	C26—C25—H25B	109.3
H10A—C10—H10B	108.5	H25A—C25—H25B	107.9
C12—C11—C10	121.43 (16)	N3—C26—C25	110.05 (14)
C16—C11—C10	120.25 (15)	N3—C26—H26A	109.6
C16—C11—C12	118.32 (16)	N3—C26—H26B	109.6
C11—C12—H12	119.7	C25—C26—H26A	109.6
C13—C12—C11	120.70 (16)	C25—C26—H26B	109.7
C13—C12—H12	119.7	H26A—C26—H26B	108.2
			
C10—O1—C1—C2	162.84 (13)	C4—C5—C6—C7	−0.7 (3)
C10—O1—C1—C17	−74.46 (17)	C8—C7—C6—C5	0.3 (3)
C1—O1—C10—C11	179.24 (13)	C9—C8—C7—C6	0.5 (3)
C25—O2—C24—C23	−59.9 (2)	C7—C8—C9—N2	178.51 (17)
C24—O2—C25—C26	60.96 (19)	C7—C8—C9—C4	−0.8 (3)
C3—N1—C2—C1	64.8 (2)	N2—C9—C4—N1	0.47 (18)
C4—N1—C2—C1	−116.02 (17)	N2—C9—C4—C5	−179.06 (15)
C2—N1—C3—N2	−179.33 (16)	C8—C9—C4—N1	179.93 (15)
C4—N1—C3—N2	1.4 (2)	C8—C9—C4—C5	0.4 (2)
C2—N1—C4—C5	−0.9 (3)	C12—C11—C10—O1	82.65 (19)
C2—N1—C4—C9	179.66 (15)	C16—C11—C10—O1	−96.93 (18)
C3—N1—C4—C5	178.42 (18)	C10—C11—C12—C13	−179.33 (16)
C3—N1—C4—C9	−1.04 (17)	C16—C11—C12—C13	0.3 (3)
C9—N2—C3—N1	−1.0 (2)	C14—C13—C12—C11	−0.7 (3)
C3—N2—C9—C4	0.32 (19)	C15—C14—C13—C12	0.9 (3)
C3—N2—C9—C8	−179.07 (18)	C13—C14—C15—C16	−0.7 (3)
C23—N3—C20—C19	−36.8 (2)	C15—C16—C11—C10	179.54 (16)
C23—N3—C20—C21	147.48 (17)	C15—C16—C11—C12	0.0 (3)
C26—N3—C20—C19	−175.07 (15)	C11—C16—C15—C14	0.3 (3)
C26—N3—C20—C21	9.2 (2)	C1—C17—C18—C19	176.97 (16)
C20—N3—C23—C24	167.07 (14)	C22—C17—C18—C19	−0.1 (3)
C26—N3—C23—C24	−52.07 (19)	C1—C17—C22—C21	−177.82 (17)
C20—N3—C26—C25	−166.46 (14)	C18—C17—C22—C21	−0.8 (3)
C23—N3—C26—C25	52.92 (19)	C20—C19—C18—C17	0.9 (3)
O1—C1—C2—N1	−62.84 (16)	N3—C20—C19—C18	−176.71 (16)
C17—C1—C2—N1	175.25 (13)	C21—C20—C19—C18	−0.8 (3)
O1—C1—C17—C18	−60.9 (2)	N3—C20—C21—C22	175.82 (16)
O1—C1—C17—C22	116.07 (17)	C19—C20—C21—C22	−0.1 (3)
C2—C1—C17—C18	58.1 (2)	C17—C22—C21—C20	0.8 (3)
C2—C1—C17—C22	−124.97 (17)	N3—C23—C24—O2	56.1 (2)
C6—C5—C4—N1	−179.01 (17)	N3—C26—C25—O2	−58.1 (2)
C6—C5—C4—C9	0.4 (2)		

### Hydrogen-bond geometry (Å, º)

Cg is the centroid of the C4–C9 benzene ring.

**Table:**

*D*—H···*A*	*D*—H	H···*A*	*D*···*A*	*D*—H···*A*
C5—H5···O2^i^	0.93	2.58	3.478 (2)	163
C6—H6···N3^ii^	0.93	2.56	3.439 (2)	159
C2—H2*A*···*Cg*^iii^	0.97	2.66	3.451 (2)	139

Symmetry codes: (i) −*x*, −*y*, −*z*; (ii) *x*, *y*, *z*+1; (iii) −*x*, −*y*+1, −*z*+1.
